# Quantifying the psychological and behavioural consequences of a diagnostic label for non-cancer conditions: systematic review

**DOI:** 10.1192/bjo.2023.49

**Published:** 2023-04-19

**Authors:** Rebecca Sims, Zoe A. Michaleff, Paul Glasziou, Mark Jones, Rae Thomas

**Affiliations:** Institute for Evidence-Based Healthcare, Bond University, Australia; Research Office, Northern New South Wales Local Health District, Australia

**Keywords:** Labelling, diagnosis, screening, consequences, systematic review

## Abstract

**Background:**

Screening for asymptomatic health conditions is perceived as mostly beneficial, with possible harms receiving little attention.

**Aims:**

To quantify proximal and longer-term consequences for individuals receiving a diagnostic label following screening for an asymptomatic, non-cancer health condition.

**Method:**

Five electronic databases were searched (inception to November 2022) for studies that recruited asymptomatic screened individuals who received or did not receive a diagnostic label. Eligible studies reported psychological, psychosocial and/or behavioural outcomes before and after screening results. Independent reviewers screened titles and abstracts, extracted data from included studies, and assessed risk of bias (Risk of Bias in Non-Randomised Studies of Interventions). Results were meta-analysed or descriptively reported.

**Results:**

Sixteen studies were included. Twelve studies addressed psychological outcomes, four studies examined behavioural outcomes and none reported psychosocial outcomes. Risk of bias was judged as low (*n* = 8), moderate (*n* = 5) or serious (*n* = 3). Immediately after receiving results, anxiety was significantly higher for individuals receiving versus not receiving a diagnostic label (mean difference −7.28, 95% CI −12.85 to −1.71). On average, anxiety increased from the non-clinical to clinical range, but returned to the non-clinical range in the longer term. No significant immediate or longer-term differences were found for depression or general mental health. Absenteeism did not significantly differ from the year before to the year after screening.

**Conclusions:**

The impacts of screening asymptomatic, non-cancer health conditions are not universally positive. Limited research exists regarding longer-term impacts. Well-designed, high-quality studies further investigating these impacts are required to assist development of protocols that minimise psychological distress following diagnosis.

## Benefits and harms of screening

Undergoing screening to identify potential health problems and risk factors is proposed as a means to improve health outcomes through early detection and treatment, increase healthy and decrease risky behaviours, and prevent premature death.^1–4^ However, in parallel with the possible benefits, screening asymptomatic individuals has the potential to construct otherwise healthy individuals as sick, and cause a substantial proportion of individuals to experience negative impacts such as psychological distress and reduced quality of life.^1–6^ Further, recent studies have suggested negative impacts in daily functioning, including losses in daily work productivity for individuals diagnosed with hypertension and heart disease.^7^ Both individuals and healthcare professionals have been found to overestimate the benefits and underestimate the harms associated with screening, with short-term reductions in psychological and psychosocial functioning reported following screening.^8–10^

Many health conditions are detected by screening asymptomatic individuals (e.g. diabetes, osteoporosis, hypertension, breast cancer and colorectal cancer).^11–15^ The impacts of cancer screening have been well researched;^16,17^ however, the impacts of screening asymptomatic non-cancer health conditions appears largely neglected. To date, research on the impacts of diagnostic labelling has predominantly focused on intervention effectiveness, including symptom management or eradication, associated stigma and/or have been conducted using hypothetical, vignette or scenario-based studies.^4,18–21^ Although important, this research overlooks the specific impact of a diagnostic label in real-world contexts.

## Benefits and harms of diagnosis

Diagnostic labels are recognised to impact an individual's understanding of self, symptoms and suffering.^22^ Labels can exaggerate perceived differences between individuals of divergent groups (e.g. those not labelled) and reduce perceived differences between individuals within similar groups (e.g. those labelled with the same diagnostic label).^22^ We recently published a scoping review that qualitatively synthesised the consequences of diagnostic labelling to develop a comprehensive framework of potential consequences following diagnostic labelling.^23^ The consequences identified were wide-ranging and both positive (positive psychological impacts, beneficial behaviour modification) and negative (negative psychological impacts, detrimental behaviour modification).^23^ How an individual incorporates the impacts of a diagnostic label can be understood through a social constructionism lens, which posits that both individual and societal factors influence understanding of, and response to, diagnostic labels.^24–26^ Given the difficulty in disentangling condition symptoms from the condition label, it is unclear whether many of the reported changes, including psychological distress and/or work absenteeism, were a result of the symptoms or the label.

## The current study

A method to disentangle symptoms from labels is to examine the consequences for asymptomatic individuals undergoing screening procedures who are, or are not, provided with a diagnostic label following screening. Although vignette and scenario-based studies provide proof of concept of the impact of a diagnostic label, longitudinal studies, preferably randomised controlled trials (RCTs), would likely provide a more accurate representation of the consequences associated with receipt of a diagnostic label. However, considering potential ethical implications of randomising individuals to receive or not receive a label, observational studies with a concurrent comparator group would also provide robust estimates of impact. The aim of this systematic review was to quantitatively synthesise the psychological, psychosocial and/or behavioural consequences for individuals receiving or not receiving a diagnostic label after being screened for an asymptomatic health condition. We aimed to describe both the proximal and longer-term impact/s of a diagnostic label following screening at one (objective 1) or more (objective 2) time points following receiving or not receiving a diagnostic label.

## Method

### Protocol and registration

The protocol for this review was registered with the International Prospective Register of Systematic Reviews (PROSPERO; identifier CRD42021261276). This review is a secondary analysis of published data and therefore did not require ethics approval. This review is reported in accordance with the Preferred Reporting Items for Systematic Reviews and Meta-Analyses (PRISMA) guidelines (for completed checklists, see Supplementary Tables 1 and 2 available at https://doi.org/10.1192/bjo.2023.49).^27^

### Eligibility criteria

We included peer-reviewed longitudinal studies with a comparator group, including RCTs, non-RCTs, and prospective and retrospective cohort studies that investigated the psychological, psychosocial and/or behavioural consequences of receiving or not receiving a diagnostic label after being screened for an asymptomatic health condition. Hereafter, individuals receiving, and those not receiving, a diagnostic label will be referred to as ‘labelled' and ‘not labelled', respectively. Given variability in terminology referring to diagnosis, the current study defined ‘labelled’ as individuals who received a test result that indicated presence or likely presence of a specific health condition, and ‘not labelled’ as individuals who received a test result that suggested no or low likelihood of presence of a specific health condition. We excluded studies reporting on cancer screening as previous systematic reviews have been conducted in this area.^16,17^ There is also evidence that suggests a cancer diagnosis, compared with a non-cancer diagnosis, can evoke a greater fear response because of the anticipated lethality of the diagnosis and preference for invasive treatments.^28–32^ Excluding studies reporting on cancer screening ensured the findings of this review could be compared with, but not influenced by, cancer conditions. We also excluded studies that used hypothetical scenarios and studies labelling individuals with intellectual disabilities and/or attributes such as race, sexual identity or sexual orientation (see Supplementary Table 3 for inclusion and exclusion criteria).

### Objective 1

For objective 1, primary studies were required to report data at two time points: pre-screening (baseline) and after receiving screening results. For psychological and psychosocial outcomes (e.g. anxiety, quality of life), close proximity of these measures to the screening results was considered important and, therefore, the second data point was required to be within 2 weeks of receiving screening results (immediate post). We hypothesised that the psychological or psychosocial impact of a label would be greatest soon after receipt of a label. The short time period also helped to minimise the impact of any treatment or management intervention. In contrast, for behavioural outcomes (i.e. employment/school absenteeism), a longer but equivalent timeframe was considered important. Therefore, retrospective cohort studies reporting routinely collected administrative data were identified and included if they reported on equivalent periods pre- and post-screening (e.g. 1 month pre/post, 1 year pre/post).

### Objective 2

Objective 2 required primary studies to report data at at least three time points: pre-screening, within 2 weeks of receiving screening results and at least one other time point thereafter. Additional time points after receiving screening results were defined as short (between 2 weeks and 3 months), medium (between 3 and 6 months) or long (between 6 and 12 months) term. To minimise the impact of treatment and further testing on either the labelled or not labelled group, primary studies were only eligible if both groups were treated and followed up equally (i.e. minimising performance bias, e.g. if additional testing or intervention was required, both groups received this). If the labelled and not labelled groups were treated differently following receipt of a diagnostic label, data were extracted up to the time point before the groups received unequal treatment.

### Information sources

Searches were conducted in PubMed, Embase, PsycINFO, Cochrane Reviews and Trials, and CINAHL from inception to 25 November 2022 (this updated a previous search conducted on 14 July 2021). We identified additional studies by reviewing the reference lists and conducted forward citation searches of included studies. Reference lists of relevant systematic reviews were examined for additional relevant studies not identified in the search.

### Search strategy and selection process

Search strategies combined medical subject headings and key word terms related to ‘diagnosis’ and ‘psychological impact’, with the original search strategies (Supplementary Table 4) revised to include additional terms related to ‘anxiety inventory’ and ‘coping’ (Supplementary Table 5). Pairs of review authors (R.S. and R.T./Z.A.M.) independently screened studies, and discrepancies were identified and resolved by discussion or in consultation with a third reviewer as necessary.

### Data extraction

Data extraction was independently completed by pairs of reviewers (R.S. and R.T./Z.A.M.). Data extracted from eligible studies included study characteristics (e.g. author, publication year, country, design, condition screened, sample size), respondent perspective (i.e. individual labelled, parent of labelled child) and participant characteristics (e.g. age, gender) and quantitative data (e.g. mean, s.d., change scores) of relevant outcomes for pre- and post-screening.

### Outcomes

Outcomes likely to be impacted by a diagnostic label were selected based on clinical relevance and the findings of previous reviews.^8,23,33,34^

#### Psychological and psychosocial outcomes

Where possible, psychological (anxiety, depression, general mental health) and psychosocial outcomes (quality of life) were extracted as total mean change scores. When total mean change scores were unavailable, subscale mean change scores were extracted. For anxiety data, state anxiety was extracted as it is suggested to be transitory compared with trait anxiety, which is considered more stable across time and situations.^35^

#### Behavioural outcomes

For behavioural outcomes (i.e. employment/school absenteeism), routinely recorded administrative data was extracted. No reliable methods of quantifying additional behavioural outcomes (e.g. physical activity) met the reviews inclusion criteria; therefore, behavioural outcomes were restricted to employment/school absenteeism.

#### Risk of bias

Risk of bias was assessed with the Risk of Bias in Non-Randomised Studies of Interventions (ROBINS-I) tool.^36^ To ensure accurate interpretation and application of the ROBINS-I tool to the current review, three authors (R.S., Z.A.M. and R.T.) independently assessed the risk of bias for three included studies, and discussed and resolved disagreements. A further three studies were assessed by two authors (R.S. and Z.A.M.) to increase rigour, with the remaining included studies assessed by one reviewer (R.S.). When required, clarification was sought from the wider research team.

### Data synthesis and analysis

Data for labelled and not labelled groups were extracted and synthesised per outcome (e.g. anxiety, depression, etc.). When clinical homogeneity existed, results were meta-analysed in RevMan 5.4.1 for Windows (Cochrane Collaboration, London, UK; https://training.cochrane.org/online-learning/core-software/revman), using mean change scores in a random and fixed (sensitivity analysis) effects model, and reported as mean difference or standardised mean difference (SMD) with s.d.^37^ Given the small number of studies and the potential lack of reliability of random-effects models when five or less studies are included, fixed-effects modes were also conducted as sensitivity analysis to increase the certainty of results.^38^ Random-effects model results are reported for all comparisons, and fixed-effects model results only if they differed. Where meta-analysis was not possible because of insufficient included studies or available data, data were reported descriptively with mean change and s.d., or ranges when s.d. could not be calculated.

Where possible, we undertook subgroup analyses to compare outcomes for individuals without a label (e.g. no diagnosis, low risk) with individuals with a label relative to their risk (e.g. moderate risk, high risk), where risk of condition is the likelihood, based on clinical indicators, of an individual developing the assessed health condition. Data examining similar outcomes were pooled, and results reported descriptively. When feasible, subgroup analyses were conducted with either meta-analyses or descriptive summaries (e.g. mean change, s.d.), to examine the contribution of diagnostic label (e.g. heart disease, osteoporosis) on outcomes.

## Results

### Study selection

**Fig. 1:**
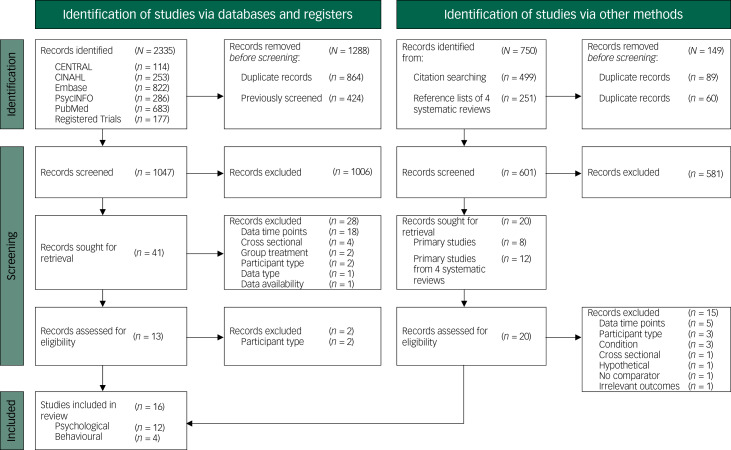
Preferred Reporting Items for Systematic Reviews and Meta-Analyses (PRISMA) flow diagram.

Searches identified 1648 unique records, of which 61 primary study full texts were retrieved and 16 primary studies were included in this systematic review (Fig. 1).

### Study characteristics

All included studies examined screening for physical health conditions (e.g. foetal abnormalities, hypertension). Ten of the 16 studies examined individual perspectives (one RCT, nine comparative observational studies) and six studies examined parent perspectives (one RCT, five comparative observational studies). All included studies met the criteria for objective 1 (12 studies reported psychological outcomes, four studies reported behavioural outcomes). Four of the 16 studies met the criteria for objective 2 (all studies reported psychological outcomes). For brevity, objectives will be referred to as objective 1 or objective 2.

Key characteristics of the included studies are reported in Table 1. Studies ranged in size from 46 to 4686 participants, and were conducted in the UK,^39–42^ USA,^43–45^ Taiwan,^46–48^ Canada,^49,50^ The Netherlands,^51,52^ Italy^53^ and Denmark,^54^ between 1977 and 2021. The included studies used different terminology to describe participants who were labelled (e.g. diagnosis, high risk, positive result, abnormal result) and were not labelled (e.g. no diagnosis, low risk, negative result, normal result). Twelve included studies reported psychological outcomes (anxiety, depression, general mental health), four included studies collected behavioural outcomes (absenteeism) and no included studies examined psychosocial outcomes (e.g. quality of life).

**Table 1:** Key characteristics of included studies

Author, year	Study type	Country	Outcome	Measure/s (objective)	Groups (*n*)	Average age in years (range)	% Female	Data collection points			Risk of bias
								Baseline	Immediate	Follow-up	
Foetal anomalies (three studies)											
Bardi et al, 2021^52^	Cohort	The Netherlands	Psychological	STAI (2)	True negative (911) False positive (26) True positive (4)	33 (18–49)	100	13 weeks’ gestation	13 weeks’ gestation	20 weeks’ gestation	Serious
Burton et al, 1985^43^	Cohort	USA	Psychological	STAI (1)	Normal result (192) Elevated result (164)	28 (not reported)	100	Before screening	<1 week	−	Moderate
Marteau et al, 1992^41^	Cohort	UK	Psychological	STAI (1)	Normal result (346) Abnormal result (26)	Not reported (<38)	100	Before screening	Post results	−	Moderate
Down syndrome (three studies)											
Cheng et al, 2008^47^	RCT	Taiwan	Psychological	STAI (1)	Negative result (2673) Positive result (109)	Not reported (not reported)	100	Before screening	3 days	−	Low
Chueh et al, 2007^48^	Cohort	Taiwan	Psychological	STAI (1)	Negative result (180) Positive result (172)	Not reported (not reported)	100	Before screening	1 week	−	Low
Quagliarini et al, 1998^53^	Cohort	Italy	Psychological	STAI (1)	Decreased risk (36) Increased risk (10)	33 (not reported) 29 (not reported)	100	11–13 weeks’ gestation	15–16 weeks’ gestation	-	Low
Group B streptococcal in pregnancy (one study)											
Cheng et al, 2006^46^	Cohort	Taiwan	Psychological	STAI (2)	Negative result (112) Positive result (71)	Not reported (not reported)	100	Before screening	1 week	1 week postpartum	Low
Heart disease (two studies)											
Connelly et al, 1998^39^	Cohort	UK	Psychological	GHQ (1) STAI (1)	Low risk (3114) Moderate risk (734) High risk (838)	Not reported (45–69)	0	Before screening	<10 days	−	Low
Jørgensen et al, 2009^54^	RCT	Denmark	Psychological	SCL-90-R (1)	Low risk (not reported) High risk (not reported)	Not reported (not reported)	Not reported	1 month before screening	<1 h	−	Moderate
Hypertension (five studies)											
Mann, 1977^40^	Cohort	UK	Psychological	GHQ (1)	Normal result (233) Recalled result (231) Elevated result (235)	Not reported (35–64)	Not reported	Before screening	Post results	−	Low
Johnston et al, 1984^49^	Cohort	Canada	Behavioural	Absenteeism (1)	Normotensive (216) Hypertensive (226)	Not reported (not reported)	0	Year before screening	Year post screening	−	Low
Rudd et al, 1987^44^	Cohort	USA	Behavioural	Absenteeism (1)	Normotensive (768) Labile hypertension (394) Sustained hypertension (294)	43 (not reported) 44 (not reported) 47 (not reported)	Not reported Not reported Not reported	Year before screening	Year post screening	−	Serious
Sexton and Schumann, 1985^45^	Cohort	USA	Behavioural	Absenteeism (1)	Normotensive (732) hypertensive (88)	Not reported (<50) Not reported (<50)	59 43	Year before screening	Year post screening	−	Serious
Stenn et al, 1981^50^	Cohort	Canada	Behavioural	Absenteeism (1)	Normotensive (72) Hypertensive (72)	Not reported (10–18)	44	Year before screening	Year post screening	−	Low
Type 2 diabetes (one study)											
Adriaanse et al, 2004^51^	Cohort	The Netherlands	Psychological	W-BQ12 (2)	No diabetes (143) Type 2 diabetes (116)	62 (not reported) 63 (not reported)	43 46	Before screening	2 weeks	6 months; 12 months	Moderate
Osteoporosis (one study)											
Rimes et al, 1999^42^	Cohort	UK	Psychological	VAS-A (2) VAS-D (2)	High result [low risk] (90) Low result [high risk] (90)	Not reported (32–73)	100	Before screening	Post results	3 months	Moderate

STAI, State Trait Anxiety Inventory; RCT, randomised controlled trial; GHQ, General Health Questionnaire; SCL-90-R, Symptom Checklist 90, revised; W-BQ12, Wellbeing Questionnaire 12 items; VAS-A, 0–100 Visual Analogue Scale measuring Anxiety; VAS-D, 0–100 Visual Analogue Scale measuring Depression.

### Risk of bias of included studies

Five of the 16 included studies were assessed to have moderate risk of bias resulting from either confounding biases (*n* = 3) or missing data (*n* = 2). Three included studies were assessed to have serious risk of bias resulting from both confounding biases and missing data. The remaining eight included studies were assessed to have low risk of bias, with detailed risk-of-bias analyses available in Supplementary Table 6.

### Outcomes

Results are reported by outcomes and study objective, with a summary of findings available in Table 2. Thresholds for clinical cut-offs for relevant measures are provided in Supplementary Table 7.

**Table 2:** Summary of findings

Outcome	Study	Measure	Objective 1: two data collection points			Objective 2: Three or more data collection points			Risk of bias
			Label	No label	Significance	Label	No label	Significance	
Meta-analysed outcomes									
Anxiety	Bardi et al, 2021^52^	STAI	↑^a^	↓	<0.001	↓	↓	0.27	Serious
	Cheng et al, 2006^46^	STAI	↑^a^	↓	<0.001	↓	↓	<0.01	Low
	Cheng et al, 2008^47^	STAI	↑^a^	↓	<0.001	−	−	−	Low
	Chueh et al, 2007^48^	STAI	↑^a^	↓	<0.001	−	−	−	Low
	Connelly et al, 1998^39^	STAI	↓	↓	0.27	−	−	−	Low
General mental health	Adriaanse et al, 2004^51^	W-BQ12	↑	↑	0.90	↑ (6 months)	↑ (6 months)	0.47	Moderate
						↓ (12 months)	↑ (12 months)	0.39	
	Connelly et al, 1998^39^	GHQ	↓	↓	0.58	−	−	−	Low
Absenteeism	Johnston et al, 1984^49^	Days	↑	↑	0.10	−	−	−	Low
	Rudd et al, 1987^44^	Episodes	↑	↑	0.53	−	−	−	Serious
	Sexton and Schumann, 1985^45^	Episodes	↑	↑	0.67	−	−	−	Serious
Narrative synthesis									
Anxiety	Burton et al, 1985^43^	STAI	↑^a^	↓	No/insufficient data reported	−	−	−	Moderate
	Marteau et al, 1992^41^	STAI	↑^a^	↓	No/insufficient data reported	−	−	−	Moderate
	Quagliarini et al, 1998^53^	STAI	↑^a^	↓	No/insufficient data reported	−	−	−	Low
	Rimes et al, 1999^42^	VAS-A	↑	↓	<0.001	↑	↓	<0.01	Moderate
	Jørgensen et al, 2009^54^	SCL-90-R(A)	↓	↓	No/insufficient data reported	−	−	−	Moderate
Depression	Rimes et al, 1999^42^	VAS-D	↓	↑	<0.001	↑	↑	0.15	Moderate
	Jørgensen et al, 2009^54^	SCL-90-R(D)	↓	↓	No/insufficient data reported	−	−	−	Moderate
General mental health	Mann 1977^40^	GHQ	No/insufficient data reported	No/insufficient data reported	No/insufficient data reported^b^	−	−	−	Low
Absenteeism	Stenn et al, 1981^50^	Days	↑	↑	No/insufficient data reported	−	−	−	Low

Significance refers to the significance value between the labelled and not labelled group; ↑ indicates that scores increased from baseline; ↓ indicates that scores decreased from baseline. STAI, State Trait Anxiety Inventory; W-BQ12, Wellbeing Questionnaire 12 items; GHQ, General Health Questionnaire; VAS-A, 0–100 Visual Analogue Scale measuring Anxiety; SCL-90-R(A), Symptom Checklist 90, revised Anxiety subscale; VAS-D, 0–100 Visual Analogue Scale measuring Depression; SCL-90-R(D), Symptom Checklist 90, revised Depression subscale.

a. Indicates scores increased to clinically significant range at immediate/longer-term follow-up for scale used.

b. Author reported no differences between labelled and not labelled from baseline to immediately following results.

### Psychological outcomes: anxiety

Ten studies measured anxiety with the State Trait Anxiety Inventory (STAI; *n* = 8),^39,41,43,46–48,52,53^ the Symptom Checklist 90 revised anxiety subscale (SCL-90-R(A); *n* = 1)^54^ and a single question about general anxiety measured on a 0–100 visual analogue scale (VAS-A; *n* = 1).^42^ Five studies^39,46–48,52^ contributed sufficient data for meta-analysis and the remaining five studies^41–43,53,54^ were narratively reported because of insufficient or non-comparable data (i.e. one question rating anxiety). Risk of bias was assessed as low (*n* = 5),^39,46–48,53^ moderate (*n* = 4)^41–43,54^ and severe (*n* = 1).^52^

#### Objective 1: changes in anxiety from baseline to immediate follow-up (*n* = 10)

**Fig. 2:**
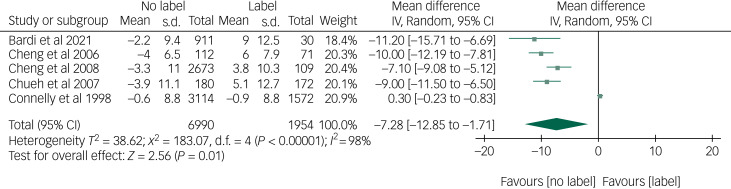
Meta-analysis of mean change in state anxiety scores from baseline to immediate follow-up. IV, inverse variance.

There was a change in anxiety from baseline to immediately after receiving screening results, with a mean difference of −7.28 (95% CI −12.85 to −1.71; Fig. 2),^39, 46–48, 52^ suggesting anxiety was reduced for individuals not labelled, and increased for individuals labelled, after receiving screening results. Given the high heterogeneity, *post hoc* sensitivity analysis was conducted, excluding the study by Connelly et al^39^ from the analysis. This reduced heterogeneity, but the overall direction and significance of effects remained unchanged (Supplementary Fig. 1). Additional *post hoc* sensitivity meta-analysis was conducted, including the study measuring anxiety with the VAS-A.^42^ For both the meta-analysis with (Supplementary Fig. 2) and without (Supplementary Fig. 3) the study by Connelly et al,^39^ the overall direction and significance of effects were unchanged.

**Fig. 3:**
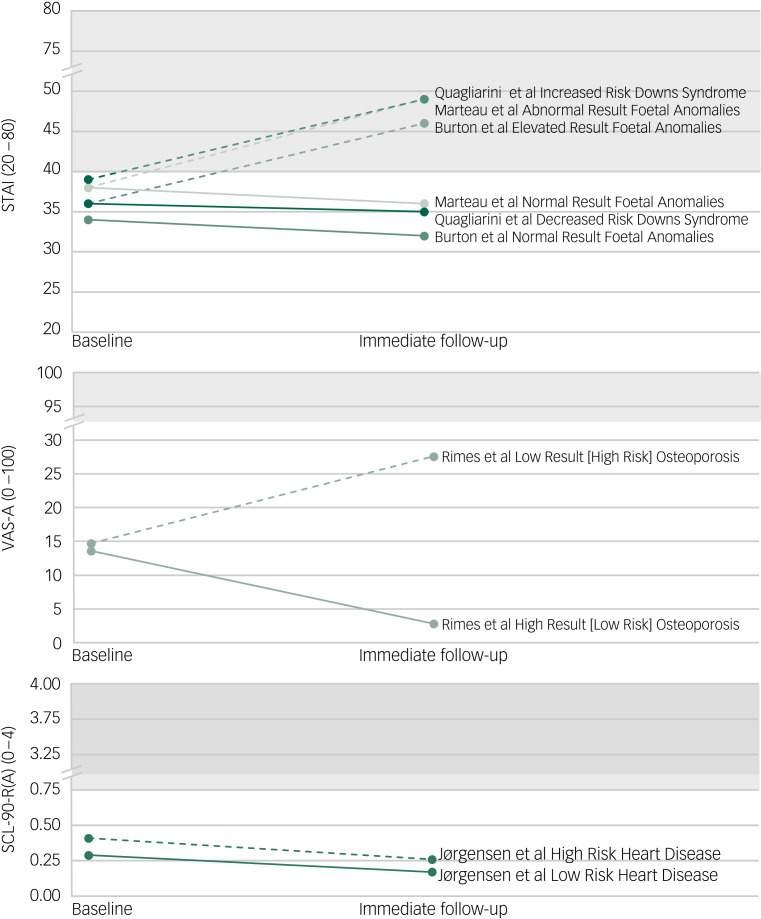
Narrative synthesis of mean change in state anxiety scores from baseline to immediate follow-up. Dashed lines represent labelled groups, solid lines represent not labelled groups and shaded areas indicate clinical range. SCL-90-R(A), Symptom Checklist 90 revised anxiety subscale; STAI, State Trait Anxiety Inventory; VAS-A, visual analogue scale – general anxiety.

Findings from the five studies^41–43,53,54^ unable to be meta-analysed supported the meta-analysis findings (Fig. 3). Specifically, all groups reported baseline anxiety within the non-clinical range. Immediately after receiving screening results, the not labelled groups reported slight reductions in anxiety. For individuals receiving a diagnostic label, anxiety increased in four studies, and in three of these, anxiety rose from the non-clinical to clinical range;^41,43,53^ however, in one study^42^ that screened for osteoporosis, although anxiety increased for those labelled, it was within the non-clinical range at both time points. Differing from other studies, results from Jørgensen et al,^54^ whose participants were screened for heart disease, suggest anxiety decreased for both groups and was consistently within the non-clinical range.

#### Objective 2: changes in anxiety from baseline to longer-term follow-up (*n* = 3)

No significant differences in anxiety between labelled and not labelled groups were found from baseline to 3 months after receiving screening results (mean difference −0.92, 95% CI −6.30 to 4.46; Supplementary Fig. 4).^46,52^ However, results of the fixed-effects meta-analysis were inconsistent with random-effects meta-analysis, and demonstrated a small but significant difference in anxiety between labelled and not labelled groups from baseline to 3 months (mean difference −2.22, 95% CI −3.78 to −0.65; Supplementary Fig. 5).^46,52^ Because of non-comparable data, findings from Rimes et al^42^ were not included in the meta-analysis, yet supported the findings from the fixed-effect model. Specifically, this study found an increase in anxiety for the labelled group (mean change 8.1, s.d. 23.9) and decrease in anxiety for the not labelled group (mean change −1.6, s.d. 18.3) from baseline to within 3 months.^42^ *Post hoc* sensitivity meta-analysis, which included the study by Rimes et al^42^ study, was conducted, with the results unchanged for both the random- (Supplementary Fig. 6) and fixed-effects (Supplementary Fig. 7) meta-analysis.

Overall, findings suggest anxiety increases immediately after being labelled; however, results for the longer-term impact are inconsistent and may be inaccurate because of the differences between random- and fixed-effects meta-analysis, the heterogeneity and limited number of studies.

### Psychological outcomes: depression

Depression was measured in two studies. One study used the SCL-90-R depression subscale (SCL-90-R(D))^54^ and the other measured general depression on a VAS (VAS-D),^42^ with both judged to have moderate risk of bias.

#### Objective 1: changes in depression from baseline to immediate follow-up (*n* = 2)

Findings from the two studies differed (Supplementary Fig. 8). Rimes et al^42^ reported that depression increased for the labelled group (mean change 6.3, s.d. 20.2) and decreased for the not labelled group (mean change −4.7, s.d. 16.7), from baseline to immediately after receiving screening results; this between-group difference was statistically significant. In contrast, Jørgensen and colleagues^54^ reported a statistically non-significant decrease in both groups following screening (not labelled: mean change −0.14; labelled: mean change −0.18; s.d. not reported). Depression scores across both studies were reported within the non-clinical range for all time points.

#### Objective 2: changes in depression from baseline to longer-term follow-up (*n* = 1)

Although not statistically significant, Rimes et al^42^ reported a short-term (from baseline to within 3 months) increase in depression scores in both labelled (mean change 7.8, s.d. 21.7) and not labelled (mean change 3.4, s.d. 18.8) groups. Depression scores were reported in the non-clinical range for both groups.

Findings for the impact of labels on depression are limited and inconsistent. Depression scores did not reach the clinical thresholds for any study.

### Psychological outcomes: general mental health

Three studies examined general mental health following screening, with risk of bias assessed as low in two studies^39,40^ and moderate in one study.^51^ Two studies used versions of the General Health Questionnaire (GHQ)^39,40^ and one study used the 12-Item Wellbeing Questionnaire (W-BQ12).^51^

#### Objective 1: changes in general mental health from baseline to immediate follow-up (*n* = 3)

No significant differences were found in general mental health scores for labelled and not labelled groups following screening (SMD = −0.02; 95% CI −0.08 to 0.04; Supplementary Fig. 9).^39,51^ Mann^40^ also reported no differences between labelled and not labelled groups from baseline to immediately following screening. However, Connelly et al^39^ reported general mental health concerns in the clinical range for both groups at both time points.

#### Objective 2: changes in general mental health from baseline to longer-term follow-up (*n* = 1)

At 6- and 12-month follow-up, Adriaanse et al^51^ reported no difference in general mental health between labelled (6-month mean change 0.8, s.d. 6.7; 12-month mean change 0.5, s.d. 6.7) and not labelled (6-month mean change 0.2, s.d. 6.2; 12-month mean change −0.2, s.d. 6.5) groups.

Although there is a consistent finding of no short- or long-term impacts of labelling on an individual's general mental health, there are few studies examining this construct and these results may be erroneous.

### Behavioural outcomes: absenteeism

Four studies^44,45,49,50^ reported on employment/school absenteeism in the year before and the year following screening. Two studies^44,45^ reported on the average number of illness episodes, regardless of length, and were assess to have moderate risk of bias, and two studies^49,50^ reported on average number of illness days and were assessed to have low risk of bias.

#### Objective 1: changes in absenteeism from one year prior to one year following (*n* = 4)

Meta-analysis suggests no significant differences in illness absenteeism in the year before and the year following screening for labelled and not labelled groups (SMD = −0.06, 95% CI −0.14 to 0.02; Supplementary Fig. 10).^44,45,49^ Similarly, Stenn et al^50^ (insufficient data for meta-analysis) reported no differences in absenteeism pre- to post-screening between labelled (mean change 2.4, s.d. not reported) and not labelled (mean change 1.3, s.d. not reported) groups.

Included studies suggest that in the year following screening, there are not significant differences in illness absenteeism for individuals receiving and not receiving a label.

### Impacts of condition severity

Four studies^39, 40, 44, 52^ provided labels that grouped individuals in different risk profiles (e.g. low-, moderate- or high-risk) following screening.

#### Anxiety (*n* = 2)

In a study investigating foetal chromosomal abnormalities, Bardi et al^52^ reported increases in anxiety immediately after receiving either a high- or moderate-risk label, with the high-risk group increasing within the clinical range and the moderate-risk group increasing from the non-clinical to clinical range; however, both groups reduced to non-clinical levels by 3 months. In contrast, a study^39^ investigating coronary heart disease reported largely unchanged anxiety for all groups, with anxiety in the non-clinical range at both time points (i.e. baseline and immediately following receiving screening results; Supplementary Fig. 1^1^). In both studies,^39,52^ anxiety remained in non-clinical range at all time points for individuals who were labelled as low risk.

#### General mental health (*n* = 2)

Connelly et al,^39^ in a study screening for heart disease, reported relatively stable general mental health from baseline to immediately following receiving a low-risk (mean change −0.3, s.d. 5.8), moderate-risk (mean change 0.2, s.d. 6.1) or high-risk (mean change −0.6, s.d. 5.5) label. However, an earlier study by Mann^40^ exploring screening for hypertension reported a deterioration in general mental health for all risk severity labels (no data reported).

#### Absenteeism (*n* = 1)

Rudd et al^44^ reported no significant between-group differences for episodes off work owing to illness in the year before to the year following receiving results of hypertension screening.

## Discussion

Comprehensive synthesis of the psychological and behavioural impacts of being labelled following screening for asymptomatic health conditions was warranted. We extracted data from 16 studies to examine the immediate and longer-term outcomes for individuals who are labelled, or not labelled, following asymptomatic screening for non-cancer health conditions. We found significant differences in anxiety in individuals who were labelled versus not labelled. Anxiety in individuals who were not labelled remained in the non-clinical range at all time points; however, anxiety in individuals who were labelled with a non-cancer diagnosis increased from the non-clinical to clinical range immediately following receipt of screening results, but returned to the non-clinical range within 3 months. In contrast, other psychological and behavioural outcomes demonstrated no significant or inconsistent change immediately, and within the longer-term, following asymptomatic screening results. Similar inconsistencies were found for stratified label use.

### Strengths and limitations

The inclusion of studies with a contemporary control group (‘not labelled’) enabled estimation of the impact of a label between individuals who were labelled and not labelled.^55^ Further, included study designs (each requiring a comparator group) investigating asymptomatic screening enabled greater disentanglement of the label impact from the impact of symptoms. *A priori* inclusion criteria required the labelled and not labelled groups to have comparable treatment and follow-up, therefore reducing potential performance bias. Investigating both immediate and longer-term impacts of a label is identified as both a strength, as we were able to demonstrate changes in psychological and behavioural outcomes over time, and a limitation, as our conclusions are limited by the paucity of research on longer-term impacts following labelling.

This review includes studies reporting on a range of health conditions and heterogeneity is expected, including possible variation in the stability of the longer-term psychological impacts. Given data availability for the current review, determination of differences across various health conditions is not currently possible; however, as more literature becomes available, this may be possible in the future. The decision to restrict included screening to non-cancer conditions potentially limits the generalisability of results. However, the omission of cancer conditions reduced potential biasing of results to known impacts of cancer condition diagnosis (e.g. fear, lethality, invasive treatment preferences)^28–32^ and provided opportunity for more accurate exploration of the impact of a label. Although potential disparities between cancer and non-cancer diagnoses exist, results of the current review are comparable to a recent systematic review on the impact of cervical cancer screening,^56^ discussed below.

### Study results in relation to other reviews

Despite these limitations, our review is similar to findings of previous reviews of both cancer and non-cancer conditions. Shaw et al^33^ conducted a systematic review (*n* = 54 studies) on the impact of predicting risk of cancer and non-cancer conditions within 4 weeks after testing. Their results suggest significant short-term increases in anxiety and depression in those testing positive, but these were not sustained in the longer term.^33^ Similarly, a systematic review by Collins et al^8^ (*n* = 12 studies) on the impacts of screening cancer and non-cancer conditions, found no significant longer-term impact of screening in these conditions. Further, a systematic review by Oliveri et al^57^ (*n* = 47 studies) found no significant increase in psychological distress following genetic testing for cardiovascular, neurodegenerative and cancer conditions, the only exception being for Huntington's disease.

Our results also align with a systematic review specific to cervical cancer screening.^56^ The systematic review by McBride et al^56^ (*n* = 33 studies) found women who received a positive label following cervical cancer screening experienced higher short-term anxiety and psychological distress, compared with those with a negative result.^56^ This short-term increase in anxiety was not sustained at 2 months. However, potentially corroborating our contention that screening for cancer conditions might have differing results, McBride et al^56^ found sustained differences in general psychological distress between individuals receiving positive and negative results.

Findings of our review related to behavioural outcomes (employment/school absenteeism) following asymptomatic non-cancer screening contrast findings from similar reviews. Two reviews, one by MacDonald et al^34^ and the other by Guirguis-Blake et al,^58^ both reported inconsistent findings related to the impact of labelling hypertension on employment absenteeism. Further, our findings pertaining to the impact of different severity labels also contrast results of an existing review,^59^ which reported different severity labels impact psychological and behavioural outcomes. Given limited studies contribute to these results, both behavioural impacts of labelling and psychological impacts of stratified diagnostic label use should not be discounted.

The results of this systematic review support those found by a qualitative review conducted by the same authors, which suggested that potential consequences following diagnostic labelling are diverse and include both positive and negative experiences.^23^ The current review also supports concepts proposed by social constructionism and modified labelling theory, which suggest multifaceted responses following diagnostic labelling.^60,61^ Further, these results support existing theories on coping with, and adjusting to, illness, which purport that adaptation and adjustment to diagnosis is possible.^62,63^ However, these latter theories suggest adjustment is confounded by multiple factors, including personal, emotional, social and healthcare systems, which was outside the scope of this systematic review.^62,63^

### Clinical implications

The findings of this review have clinical and practical implications. Primarily, because of the general increase in anxiety (at times to within the clinical range) for individuals labelled immediately after receiving screening results, it identifies the need for clinicians to integrate patient education and decision aids related to potential increase in psychological distress before screening. Such practices may provide patients with necessary information (e.g. benefits and harms) to more actively participate in shared decision-making and minimise psychological distress. More informed patients and decisions may result in a decrease in psychological and behavioural distress following labelling.^64,65^ Additionally, routine collection of patient-reported outcome measures (PROMs) and patient-reported experience measures (PREMs) will assist in monitoring, and further quantifying, the impact of screening.^66^ Incorporating adequate test characteristics, healthcare professional and patient discussion, and patient monitoring could alleviate, or reduce the intensity of, possible psychological and behavioural distress resulting from diagnostic labelling following asymptomatic screening.

### Future research

This review highlighted several areas for additional research. Although this review examined psychological and behavioural impacts of labelling following screening for asymptomatic non-cancer conditions, examining the impact of labelling in other scenarios, such as incidental diagnoses (e.g. diagnosis of a condition found during testing for a different condition) and/or symptomatic conditions, will elicit similarities and differences in varied diagnostic contexts. Similarly, broadening the understanding of the impacts of labelling across a wider range of diagnostic labels, including psychological labels, will provide insight into the applicability of the current results to different diagnoses. To support clinical practice, additional research into, and/or development of, decision aids, and selection of the most appropriate PROMs and PREMs, to support screening practices and programmes is required.

The findings of this systematic review suggest that screening is not universally positive. Some individuals receiving a diagnostic label experience clinical levels of anxiety immediately on hearing this news. Although this appears transient, there are few high-quality, well-designed studies that measure the short-, medium- or long-term impacts of a diagnostic label. So, we cannot be certain. Before screening, discussion of the potential harms and benefits with individuals, and balancing individual informed decisions and clinical indication, should occur. Additional research, using rigorous methodologies, exploring the quantifiable impacts following diagnostic labelling and including diverse diagnostic contexts, is also required.

## Data Availability

Data generated and/or analysed during the current study are available from the corresponding author, R.S., on reasonable request.

## Supplementary material

For supplementary material accompanying this paper visit http://doi.org/10.1192/bjo.2023.49. click here to view supplementary material
