# Supplementary material for: Quantifying the psychological and behavioural consequences of a diagnostic label for non-cancer conditions: systematic review
Source: BJPsych Open. 2023 Apr 19;9(3):e73. doi: 10.1192/bjo.2023.49 (PMC10134215; doi:10.1192/bjo.2023.49)
Supplement: Supplementary file 1 [file S2056472423000492sup001.docx]

**Supplementary Files**

Supplementary Table 1. PRISMA 2020 checklist.

| **Section and Topic** | **Item #** | **Checklist item** | **Location where item is reported** |
| --- | --- | --- | --- |
| **TITLE** | | |  |
| Title | 1 | Identify the report as a systematic review. | 1 |
| **ABSTRACT** | | |  |
| Abstract | 2 | See the PRISMA 2020 for Abstracts checklist. | Supp Table 2 |
| **INTRODUCTION** | | |  |
| Rationale | 3 | Describe the rationale for the review in the context of existing knowledge. | 5 |
| Objectives | 4 | Provide an explicit statement of the objective(s) or question(s) the review addresses. | 5 |
| **METHODS** | | |  |
| Eligibility criteria | 5 | Specify the inclusion and exclusion criteria for the review and how studies were grouped for the syntheses. | 6-7 and Supp Table 3 |
| Information sources | 6 | Specify all databases, registers, websites, organisations, reference lists and other sources searched or consulted to identify studies. Specify the date when each source was last searched or consulted. | 8 |
| Search strategy | 7 | Present the full search strategies for all databases, registers and websites, including any filters and limits used. | Supp Table 5 |
| Selection process | 8 | Specify the methods used to decide whether a study met the inclusion criteria of the review, including how many reviewers screened each record and each report retrieved, whether they worked independently, and if applicable, details of automation tools used in the process. | 8 |
| Data collection process | 9 | Specify the methods used to collect data from reports, including how many reviewers collected data from each report, whether they worked independently, any processes for obtaining or confirming data from study investigators, and if applicable, details of automation tools used in the process. | 8 |
| Data items | 10a | List and define all outcomes for which data were sought. Specify whether all results that were compatible with each outcome domain in each study were sought (e.g. for all measures, time points, analyses), and if not, the methods used to decide which results to collect. | 9-10 |
|  | 10b | List and define all other variables for which data were sought (e.g. participant and intervention characteristics, funding sources). Describe any assumptions made about any missing or unclear information. | 8-10 |
| Study risk of bias assessment | 11 | Specify the methods used to assess risk of bias in the included studies, including details of the tool(s) used, how many reviewers assessed each study and whether they worked independently, and if applicable, details of automation tools used in the process. | 9 |
| Effect measures | 12 | Specify for each outcome the effect measure(s) (e.g. risk ratio, mean difference) used in the synthesis or presentation of results. | 10 |
| Synthesis methods | 13a | Describe the processes used to decide which studies were eligible for each synthesis (e.g. tabulating the study intervention characteristics and comparing against the planned groups for each synthesis (item #5)). | 10 |
|  | 13b | Describe any methods required to prepare the data for presentation or synthesis, such as handling of missing summary statistics, or data conversions. | 10 |
|  | 13c | Describe any methods used to tabulate or visually display results of individual studies and syntheses. | 10 |
|  | 13d | Describe any methods used to synthesize results and provide a rationale for the choice(s). If meta-analysis was performed, describe the model(s), method(s) to identify the presence and extent of statistical heterogeneity, and software package(s) used. | 10 |
|  | 13e | Describe any methods used to explore possible causes of heterogeneity among study results (e.g. subgroup analysis, meta-regression). | 10 |
|  | 13f | Describe any sensitivity analyses conducted to assess robustness of the synthesized results. | 10 |
| Reporting bias assessment | 14 | Describe any methods used to assess risk of bias due to missing results in a synthesis (arising from reporting biases). | 9 |
| Certainty assessment | 15 | Describe any methods used to assess certainty (or confidence) in the body of evidence for an outcome. | N/A |
| **RESULTS** | | |  |
| Study selection | 16a | Describe the results of the search and selection process, from the number of records identified in the search to the number of studies included in the review, ideally using a flow diagram. | 10 and Figure 1 |
|  | 16b | Cite studies that might appear to meet the inclusion criteria, but which were excluded, and explain why they were excluded. | Figure 1 |
| Study characteristics | 17 | Cite each included study and present its characteristics. | Table 1 |
| Risk of bias in studies | 18 | Present assessments of risk of bias for each included study. | Supp Table 6 |
| Results of individual studies | 19 | For all outcomes, present, for each study: (a) summary statistics for each group (where appropriate) and (b) an effect estimate and its precision (e.g. confidence/credible interval), ideally using structured tables or plots. | Table 2 |
| Results of syntheses | 20a | For each synthesis, briefly summarise the characteristics and risk of bias among contributing studies. | 12-17 |
|  | 20b | Present results of all statistical syntheses conducted. If meta-analysis was done, present for each the summary estimate and its precision (e.g. confidence/credible interval) and measures of statistical heterogeneity. If comparing groups, describe the direction of the effect. | 12-17 |
|  | 20c | Present results of all investigations of possible causes of heterogeneity among study results. | 12-17 |
|  | 20d | Present results of all sensitivity analyses conducted to assess the robustness of the synthesized results. | 12-17 |
| Reporting biases | 21 | Present assessments of risk of bias due to missing results (arising from reporting biases) for each synthesis assessed. | Supp Table 6 |
| Certainty of evidence | 22 | Present assessments of certainty (or confidence) in the body of evidence for each outcome assessed. | N/A |
| **DISCUSSION** | | |  |
| Discussion | 23a | Provide a general interpretation of the results in the context of other evidence. | 19-20 |
|  | 23b | Discuss any limitations of the evidence included in the review. | 18 |
|  | 23c | Discuss any limitations of the review processes used. | 18 |
|  | 23d | Discuss implications of the results for practice, policy, and future research. | 20-21 |
| **OTHER INFORMATION** | | |  |
| Registration and protocol | 24a | Provide registration information for the review, including register name and registration number, or state that the review was not registered. | 6 |
|  | 24b | Indicate where the review protocol can be accessed, or state that a protocol was not prepared. | 6 |
|  | 24c | Describe and explain any amendments to information provided at registration or in the protocol. | N/A |
| Support | 25 | Describe sources of financial or non-financial support for the review, and the role of the funders or sponsors in the review. | 22 |
| Competing interests | 26 | Declare any competing interests of review authors. | 22 |
| Availability of data, code and other materials | 27 | Report which of the following are publicly available and where they can be found: template data collection forms; data extracted from included studies; data used for all analyses; analytic code; any other materials used in the review. | 22 |

Supplementary Table 2. PRISMA 2020 abstract checklist.

| **Section and Topic** | **Item #** | **Checklist item** | **Reported (Yes/No)** |
| --- | --- | --- | --- |
| **TITLE** | | |  |
| Title | 1 | Identify the report as a systematic review. | Yes |
| **BACKGROUND** | | |  |
| Objectives | 2 | Provide an explicit statement of the main objective(s) or question(s) the review addresses. | Yes |
| **METHODS** | | |  |
| Eligibility criteria | 3 | Specify the inclusion and exclusion criteria for the review. | Yes |
| Information sources | 4 | Specify the information sources (e.g. databases, registers) used to identify studies and the date when each was last searched. | Yes |
| Risk of bias | 5 | Specify the methods used to assess risk of bias in the included studies. | Yes |
| Synthesis of results | 6 | Specify the methods used to present and synthesise results. | Yes |
| **RESULTS** | | |  |
| Included studies | 7 | Give the total number of included studies and participants and summarise relevant characteristics of studies. | Yes |
| Synthesis of results | 8 | Present results for main outcomes, preferably indicating the number of included studies and participants for each. If meta-analysis was done, report the summary estimate and confidence/credible interval. If comparing groups, indicate the direction of the effect (i.e. which group is favoured). | Yes |
| **DISCUSSION** | | |  |
| Limitations of evidence | 9 | Provide a brief summary of the limitations of the evidence included in the review (e.g. study risk of bias, inconsistency and imprecision). | Yes |
| Interpretation | 10 | Provide a general interpretation of the results and important implications. | Yes |
| **OTHER** | | |  |
| Funding | 11 | Specify the primary source of funding for the review. **(At end of article)** | Yes |
| Registration | 12 | Provide the register name and registration number. **(At end of article)** | Yes |

Supplementary Table 3. Inclusion and exclusion criteria.

| **Aspect** | **Inclusion Criteria** | **Exclusion Criteria** |
| --- | --- | --- |
| Study Types | Original, peer reviewed, prospective and retrospective studies   1. RCT 2. Non-RCT 3. Prospective cohort with comparator 4. Retrospective cohort with comparator | Protocols (final study to be sourced)  Opinion pieces and commentaries  Cross-Sectional studies  Hypothetical or vignette-based studies |
| Population | Asymptomatic individuals, with no age limit (e.g., adults, children) who undergo screening for a health condition | Symptomatic individuals undertaking tests seeking potential diagnoses  Screening of cancer conditions |
| Intervention | Receipt of a health condition label following a screening test. | - |
| Comparator | Receiving no label, or a label indicating normal health following a screening test. | - |
| Outcomes | Psychological:   - Anxiety - Depression - General mental health   Psychosocial   - Quality of life   Behavioural   - Absenteeism |  |
| Timeframes | Objective 1 (immediate)   - Minimum of two time points (baseline and immediate; equivalent pre/post period for retrospective studies)   Objective 2 (over time)   - Three or more time points (e.g., baseline, immediately post, follow up) | - |
| Language | No language limitations | - |
| Date | No date limitations | - |

Supplementary Table 4. Original search strategies.

| **Database** | **Search Strategy** |
| --- | --- |
| Cochrane | ((([mh /DI] OR Labelling:ti,ab OR Labeling:ti,ab OR Classified:ti,ab OR Positively:ti,ab OR Diagnosis:ti,ab OR Detected:ti,ab OR Detection:ti,ab)  AND  ([mh "Mass Screening"] OR Screening:ti,ab OR Screened:ti,ab)  AND  ("Psychological distress":ti,ab OR "Psychological impact":ti,ab OR "Psychological effects":ti,ab OR "Anxiety levels":ti,ab OR "Mental distress":ti,ab OR Absenteeism:ti,ab)  AND  (Patient:ti,ab OR Patients:ti,ab OR Individuals:ti,ab OR Self:ti,ab OR Parent:ti,ab OR Family:ti,ab OR Adult:ti,ab OR Men:ti,ab OR Women:ti,ab OR Children:ti,ab OR Subjects:ti,ab)  AND  ([mh Attitude] OR Stigma:ti,ab OR Beliefs:ti,ab OR Well-being:ti,ab OR Wellbeing:ti,ab OR Influence:ti,ab OR Emotion:ti,ab OR Distress:ti,ab OR Mood:ti,ab OR Consequences:ti,ab OR Effect:ti,ab OR Effects:ti,ab)  AND  (Before:ti,ab OR After:ti,ab))  OR  ((Labelling:ti OR Labeling:ti OR Detection:ti) AND (Phenomenon:ti OR Psychological:ti OR [mh Absenteeism] OR Absenteeism:ti)))  AND  ("randomized controlled trial":pt OR "controlled clinical trial":pt OR randomized:ti,ab OR randomised:ti,ab OR placebo:ti,ab OR randomly:ti,ab OR trial:ti,ab OR groups:ti,ab OR [mh "Epidemiologic Studies"] OR [mh "case-control studies"] OR [mh "Cohort Studies"] OR "case control":ti,ab OR Cohort:ti,ab OR "Follow up":ti,ab OR Observational:ti,ab OR Longitudinal:ti,ab OR Prospective:ti,ab OR Retrospective:ti,ab OR Investigated:ti,ab OR Analysis:ti,ab OR Statistics:ti,ab OR Data:ti,ab OR [mh /SN] OR [mh /EP] OR Study:ti)  NOT  ([mh Animals] NOT ([mh Animals] AND [mh Humans]))  NOT  ("Systematic review":ti,ab OR "Systematic Review":pt OR "Cochrane Database Syst Rev":so OR "meta analysis":pt OR "Meta analysis":ti,ab OR Meta-analysis:ti,ab)  NOT  ([mh Injections] OR [mh Neoplasms] OR Open-Label:ti,ab OR [mh "Product Labeling"] OR [mh "Drug Labeling"] OR [mh "Affinity Labels"] OR [mh "Food Labeling"] OR [mh "Isotope Labeling"] OR [mh "Staining and Labeling"] OR [mh "In Situ Nick-End Labeling"] OR [mh "Primed In Situ Labeling"] OR Placebo:ti OR [mh /DE] OR Drug:ti OR Drugs:ti OR "Food and Drug Administration":ti OR "Food labeling":ti OR "Calorie labeling":ti OR Injection:ti OR Cigarette:ti OR Cancer:ti OR Cancers:ti) |
| CINHAL | ((("Diagnosis" OR (TI Labelling OR AB Labelling) OR (TI Labeling OR AB Labeling) OR (TI Classified OR AB Classified) OR (TI Positively OR AB Positively) OR (TI Diagnosis OR AB Diagnosis) OR (TI Detected OR AB Detected) OR (TI Detection OR AB Detection))  AND  ((MH "Mass Screening+") OR (TI Screening OR AB Screening) OR (TI Screened OR AB Screened))  AND  ((TI "Psychological distress" OR AB "Psychological distress") OR (TI "Psychological impact" OR AB "Psychological impact") OR (TI "Psychological effects" OR AB "Psychological effects") OR (TI "Anxiety levels" OR AB "Anxiety levels") OR (TI "Mental distress" OR AB "Mental distress") OR (TI Absenteeism OR AB Absenteeism))  AND  ((TI Patient OR AB Patient) OR (TI Patients OR AB Patients) OR (TI Individuals OR AB Individuals) OR (TI Self OR AB Self) OR (TI Parent OR AB Parent) OR (TI Family OR AB Family) OR (TI Adult OR AB Adult) OR (TI Men OR AB Men) OR (TI Women OR AB Women) OR (TI Children OR AB Children) OR (TI Subjects OR AB Subjects))  AND  ((MH Attitude+) OR (TI Stigma OR AB Stigma) OR (TI Beliefs OR AB Beliefs) OR (TI Well-being OR AB Well-being) OR (TI Wellbeing OR AB Wellbeing) OR (TI Influence OR AB Influence) OR (TI Emotion OR AB Emotion) OR (TI Distress OR AB Distress) OR (TI Mood OR AB Mood) OR (TI Consequences OR AB Consequences) OR (TI Effect OR AB Effect) OR (TI Effects OR AB Effects))  AND  ((TI Before OR AB Before) OR (TI After OR AB After)))  OR  (((TI Labelling) OR (TI Labeling) OR (TI Detection)) AND ((TI Phenomenon) OR (TI Psychological) OR (MH Absenteeism+) OR (TI Absenteeism))))  AND  ((PT "randomized controlled trial") OR (PT "controlled clinical trial") OR (TI randomized OR AB randomized) OR (TI randomised OR AB randomised) OR (TI placebo OR AB placebo) OR (TI randomly OR AB randomly) OR (TI trial OR AB trial) OR (TI groups OR AB groups) OR (MH "Epidemiologic Studies+") OR (MH "case-control studies+") OR (MH "Cohort Studies+") OR (TI "case control" OR AB "case control") OR (TI Cohort OR AB Cohort) OR (TI "Follow up" OR AB "Follow up") OR (TI Observational OR AB Observational) OR (TI Longitudinal OR AB Longitudinal) OR (TI Prospective OR AB Prospective) OR (TI Retrospective OR AB Retrospective) OR (TI Investigated OR AB Investigated) OR (TI Analysis OR AB Analysis) OR (TI Statistics OR AB Statistics) OR (TI Data OR AB Data) OR "Statistics & Numerical Data" OR "Epidemiology" OR (TI Study))  NOT  ((MH Animals+) NOT ((MH Animals+) AND (MH Humans+)))  NOT  ((TI "Systematic review" OR AB "Systematic review") OR (PT "Systematic Review") OR (SO "Cochrane Database Syst Rev" OR ST "Cochrane Database Syst Rev" OR IB "Cochrane Database Syst Rev") OR (PT "meta analysis") OR (TI "Meta analysis" OR AB "Meta analysis") OR (TI Meta-analysis OR AB Meta-analysis))  NOT  ((MH Injections+) OR (MH Neoplasms+) OR (TI Open-Label OR AB Open-Label) OR (MH "Product Labeling+") OR (MH "Drug Labeling+") OR (MH "Affinity Labels+") OR (MH "Food Labeling+") OR (MH "Isotope Labeling+") OR (MH "Staining and Labeling+") OR (MH "In Situ Nick-End Labeling+") OR (MH "Primed In Situ Labeling+") OR (TI Placebo) OR "Drug Effects" OR (TI Drug) OR (TI Drugs) OR (TI "Food and Drug Administration") OR (TI "Food labeling") OR (TI "Calorie labeling") OR (TI Injection) OR (TI Cigarette) OR (TI Cancer) OR (TI Cancers)) |
| Embase | ((("Diagnosis" OR Labelling:ti,ab OR Labeling:ti,ab OR Classified:ti,ab OR Positively:ti,ab OR Diagnosis:ti,ab OR Detected:ti,ab OR Detection:ti,ab)  AND  ('Mass Screening'/exp OR Screening:ti,ab OR Screened:ti,ab)  AND  ('Psychological distress':ti,ab OR 'Psychological impact':ti,ab OR 'Psychological effects':ti,ab OR 'Anxiety levels':ti,ab OR 'Mental distress':ti,ab OR Absenteeism:ti,ab)  AND  (Patient:ti,ab OR Patients:ti,ab OR Individuals:ti,ab OR Self:ti,ab OR Parent:ti,ab OR Family:ti,ab OR Adult:ti,ab OR Men:ti,ab OR Women:ti,ab OR Children:ti,ab OR Subjects:ti,ab)  AND  (Attitude/exp OR Stigma:ti,ab OR Beliefs:ti,ab OR Well-being:ti,ab OR Wellbeing:ti,ab OR Influence:ti,ab OR Emotion:ti,ab OR Distress:ti,ab OR Mood:ti,ab OR Consequences:ti,ab OR Effect:ti,ab OR Effects:ti,ab)  AND  (Before:ti,ab OR After:ti,ab))  OR  ((Labelling:ti OR Labeling:ti OR Detection:ti) AND (Phenomenon:ti OR Psychological:ti OR Absenteeism/exp OR Absenteeism:ti)))  AND  (term:it OR term:it OR randomized:ti,ab OR randomised:ti,ab OR placebo:ti,ab OR randomly:ti,ab OR trial:ti,ab OR groups:ti,ab OR 'Epidemiologic Studies'/exp OR 'case-control studies'/exp OR 'Cohort Studies'/exp OR 'case control':ti,ab OR Cohort:ti,ab OR 'Follow up':ti,ab OR Observational:ti,ab OR Longitudinal:ti,ab OR Prospective:ti,ab OR Retrospective:ti,ab OR Investigated:ti,ab OR Analysis:ti,ab OR Statistics:ti,ab OR Data:ti,ab OR "Statistics & Numerical Data" OR "Epidemiology" OR Study:ti)  NOT  (Animals/exp NOT (Animals/exp AND Humans/exp))  NOT  ('Systematic review':ti,ab OR term:it OR 'Cochrane Database Syst Rev':jt OR term:it OR 'Meta analysis':ti,ab OR Meta-analysis:ti,ab)  NOT  (Injections/exp OR Neoplasms/exp OR Open-Label:ti,ab OR 'Product Labeling'/exp OR 'Drug Labeling'/exp OR 'Affinity Labels'/exp OR 'Food Labeling'/exp OR 'Isotope Labeling'/exp OR 'Staining and Labeling'/exp OR 'In Situ Nick-End Labeling'/exp OR 'Primed In Situ Labeling'/exp OR Placebo:ti OR "Drug Effects" OR Drug:ti OR Drugs:ti OR 'Food and Drug Administration':ti OR 'Food labeling':ti OR 'Calorie labeling':ti OR Injection:ti OR Cigarette:ti OR Cancer:ti OR Cancers:ti) |
| PsycINFO | ((("Diagnosis" OR Labelling.ti,ab. OR Labeling.ti,ab. OR Classified.ti,ab. OR Positively.ti,ab. OR Diagnosis.ti,ab. OR Detected.ti,ab. OR Detection.ti,ab.)  AND  (exp "Mass Screening"/ OR Screening.ti,ab. OR Screened.ti,ab.)  AND  ("Psychological distress".ti,ab. OR "Psychological impact".ti,ab. OR "Psychological effects".ti,ab. OR "Anxiety levels".ti,ab. OR "Mental distress".ti,ab. OR Absenteeism.ti,ab.)  AND  (Patient.ti,ab. OR Patients.ti,ab. OR Individuals.ti,ab. OR Self.ti,ab. OR Parent.ti,ab. OR Family.ti,ab. OR Adult.ti,ab. OR Men.ti,ab. OR Women.ti,ab. OR Children.ti,ab. OR Subjects.ti,ab.)  AND  (exp Attitude/ OR Stigma.ti,ab. OR Beliefs.ti,ab. OR Well-being.ti,ab. OR Wellbeing.ti,ab. OR Influence.ti,ab. OR Emotion.ti,ab. OR Distress.ti,ab. OR Mood.ti,ab. OR Consequences.ti,ab. OR Effect.ti,ab. OR Effects.ti,ab.)  AND  (Before.ti,ab. OR After.ti,ab.))  OR  ((Labelling.ti. OR Labeling.ti. OR Detection.ti.) AND (Phenomenon.ti. OR Psychological.ti. OR exp Absenteeism/ OR Absenteeism.ti.)))  AND  ("randomized controlled trial".pt. OR "controlled clinical trial".pt. OR randomized.ti,ab. OR randomised.ti,ab. OR placebo.ti,ab. OR randomly.ti,ab. OR trial.ti,ab. OR groups.ti,ab. OR exp "Epidemiologic Studies"/ OR exp "case-control studies"/ OR exp "Cohort Studies"/ OR "case control".ti,ab. OR Cohort.ti,ab. OR "Follow up".ti,ab. OR Observational.ti,ab. OR Longitudinal.ti,ab. OR Prospective.ti,ab. OR Retrospective.ti,ab. OR Investigated.ti,ab. OR Analysis.ti,ab. OR Statistics.ti,ab. OR Data.ti,ab. OR "Statistics & Numerical Data" OR "Epidemiology" OR Study.ti.)  NOT  (exp Animals/ NOT (exp Animals/ AND exp Humans/))  NOT  ("Systematic review".ti,ab. OR "Systematic Review".pt. OR "Cochrane Database Syst Rev".jn,jw,is,it. OR "meta analysis".pt. OR "Meta analysis".ti,ab. OR Meta-analysis.ti,ab.)  NOT  (exp Injections/ OR exp Neoplasms/ OR Open-Label.ti,ab. OR exp "Product Labeling"/ OR exp "Drug Labeling"/ OR exp "Affinity Labels"/ OR exp "Food Labeling"/ OR exp "Isotope Labeling"/ OR exp "Staining and Labeling"/ OR exp "In Situ Nick-End Labeling"/ OR exp "Primed In Situ Labeling"/ OR Placebo.ti. OR "Drug Effects" OR Drug.ti. OR Drugs.ti. OR "Food and Drug Administration".ti. OR "Food labeling".ti. OR "Calorie labeling".ti. OR Injection.ti. OR Cigarette.ti. OR Cancer.ti. OR Cancers.ti.) |
| PubMed | ((("Diagnosis"[sh] OR Labelling[tiab] OR Labeling[tiab] OR Classified[tiab] OR Positively[tiab] OR Diagnosis[tiab] OR Detected[tiab] OR Detection[tiab])  AND  ("Mass Screening"[Mesh] OR Screening[tiab] OR Screened[tiab])  AND  ("Psychological distress"[tiab] OR "Psychological impact"[tiab] OR "Psychological effects"[tiab] OR "Anxiety levels"[tiab] OR "Mental distress"[tiab] OR Absenteeism[tiab])  AND  (Patient[tiab] OR Patients[tiab] OR Individuals[tiab] OR Self[tiab] OR Parent[tiab] OR Family[tiab] OR Adult[tiab] OR Men[tiab] OR Women[tiab] OR Children[tiab] OR Subjects[tiab])  AND  (Attitude[Mesh] OR Stigma[tiab] OR Beliefs[tiab] OR Well-being[tiab] OR Wellbeing[tiab] OR Influence[tiab] OR Emotion[tiab] OR Distress[tiab] OR Mood[tiab] OR Consequences[tiab] OR Effect[tiab] OR Effects[tiab])  AND  (Before[tiab] OR After[tiab]))  OR  ((Labelling[ti] OR Labeling[ti] OR Detection[ti]) AND (Phenomenon[ti] OR Psychological[ti] OR Absenteeism[Mesh] OR Absenteeism[ti])))  AND  ("randomized controlled trial"[pt] OR "controlled clinical trial"[pt] OR randomized[tiab] OR randomised[tiab] OR placebo[tiab] OR randomly[tiab] OR trial[tiab] OR groups[tiab] OR "Epidemiologic Studies"[Mesh] OR "case-control studies"[Mesh] OR "Cohort Studies"[Mesh] OR "case control"[tiab] OR Cohort[tiab] OR "Follow up"[tiab] OR Observational[tiab] OR Longitudinal[tiab] OR Prospective[tiab] OR Retrospective[tiab] OR Investigated[tiab] OR Analysis[tiab] OR Statistics[tiab] OR Data[tiab] OR "Statistics & Numerical Data"[sh] OR "Epidemiology"[sh] OR Study[ti])  NOT  (Animals[Mesh] NOT (Animals[Mesh] AND Humans[Mesh]))  NOT  ("Systematic review"[tiab] OR "Systematic Review"[pt] OR "Cochrane Database Syst Rev"[TA] OR "meta analysis"[pt] OR "Meta analysis"[tiab] OR Meta-analysis[tiab])  NOT  (Injections[Mesh] OR Neoplasms[Mesh] OR Open-Label[tiab] OR "Product Labeling"[Mesh] OR "Drug Labeling"[Mesh] OR "Affinity Labels"[Mesh] OR "Food Labeling"[Mesh] OR "Isotope Labeling"[Mesh] OR "Staining and Labeling"[Mesh] OR "In Situ Nick-End Labeling"[Mesh] OR "Primed In Situ Labeling"[Mesh] OR Placebo[ti] OR "Drug Effects"[sh] OR Drug[ti] OR Drugs[ti] OR "Food and Drug Administration"[ti] OR "Food labeling"[ti] OR "Calorie labeling"[ti] OR Injection[ti] OR Cigarette[ti] OR Cancer[ti] OR Cancers[ti]) |

Supplementary Table 5. Updated search strategies.

| **Database** | **Search Strategy** |
| --- | --- |
| Cochrane | ((([mh /DI] OR Diagnostic:ti,ab OR Labelling:ti,ab OR Labeling:ti,ab OR Classified:ti,ab OR Positively:ti,ab OR Diagnosis:ti,ab OR Detected:ti,ab OR Detection:ti,ab OR Scan:ti,ab)  AND  ([mh "Mass Screening"] OR Screening:ti,ab OR Screened:ti,ab)  AND  ("Psychological distress":ti,ab OR "Psychological impact":ti,ab OR "Psychological effects":ti,ab OR "Anxiety levels":ti,ab OR "Anxiety Inventory" OR STAI:ti,ab OR "Mental distress":ti,ab OR Absenteeism:ti,ab)  AND  (Patient:ti,ab OR Patients:ti,ab OR Individuals:ti,ab OR Self:ti,ab OR Parent:ti,ab OR Family:ti,ab OR Adult:ti,ab OR Men:ti,ab OR Women:ti,ab OR Children:ti,ab OR Subjects:ti,ab)  AND  ([mh Attitude] OR Stigma:ti,ab OR Beliefs:ti,ab OR Well-being:ti,ab OR Wellbeing:ti,ab OR Influence:ti,ab OR Emotion:ti,ab OR Distress:ti,ab OR Mood:ti,ab OR Consequences:ti,ab OR Effect:ti,ab OR Effects:ti,ab OR Coping:ti,ab)  AND  (Before:ti,ab OR After:ti,ab OR Following:ti,ab))  OR  ((Labelling:ti OR Labeling:ti OR Detection:ti) AND (Phenomenon:ti OR Psychological:ti OR [mh Absenteeism] OR Absenteeism:ti)))  NOT  ([mh Injections] OR [mh Neoplasms] OR Open-Label:ti,ab OR [mh "Product Labeling"] OR [mh "Drug Labeling"] OR [mh "Affinity Labels"] OR [mh "Food Labeling"] OR [mh "Isotope Labeling"] OR [mh "Staining and Labeling"] OR [mh "In Situ Nick-End Labeling"] OR [mh "Primed In Situ Labeling"] OR Placebo:ti OR [mh /DE] OR Drug:ti OR Drugs:ti OR "Food and Drug Administration":ti OR "Food labeling":ti OR "Calorie labeling":ti OR Injection:ti OR Cigarette:ti OR Cancer:ti OR Cancers:ti) |
| CINHAL | ((("Diagnosis" OR (TI Diagnostic OR AB Diagnostic) OR (TI Labelling OR AB Labelling) OR (TI Labeling OR AB Labeling) OR (TI Classified OR AB Classified) OR (TI Positively OR AB Positively) OR (TI Diagnosis OR AB Diagnosis) OR (TI Detected OR AB Detected) OR (TI Detection OR AB Detection) OR (TI Scan OR AB Scan))  AND  ((MH "Mass Screening+") OR (TI Screening OR AB Screening) OR (TI Screened OR AB Screened))  AND  ((TI "Psychological distress" OR AB "Psychological distress") OR (TI "Psychological impact" OR AB "Psychological impact") OR (TI "Psychological effects" OR AB "Psychological effects") OR (TI "Anxiety levels" OR AB "Anxiety levels") OR "Anxiety Inventory" OR (TI STAI OR AB STAI) OR (TI "Mental distress" OR AB "Mental distress") OR (TI Absenteeism OR AB Absenteeism))  AND  ((TI Patient OR AB Patient) OR (TI Patients OR AB Patients) OR (TI Individuals OR AB Individuals) OR (TI Self OR AB Self) OR (TI Parent OR AB Parent) OR (TI Family OR AB Family) OR (TI Adult OR AB Adult) OR (TI Men OR AB Men) OR (TI Women OR AB Women) OR (TI Children OR AB Children) OR (TI Subjects OR AB Subjects))  AND  ((MH Attitude+) OR (TI Stigma OR AB Stigma) OR (TI Beliefs OR AB Beliefs) OR (TI Well-being OR AB Well-being) OR (TI Wellbeing OR AB Wellbeing) OR (TI Influence OR AB Influence) OR (TI Emotion OR AB Emotion) OR (TI Distress OR AB Distress) OR (TI Mood OR AB Mood) OR (TI Consequences OR AB Consequences) OR (TI Effect OR AB Effect) OR (TI Effects OR AB Effects) OR (TI Coping OR AB Coping))  AND  ((TI Before OR AB Before) OR (TI After OR AB After) OR (TI Following OR AB Following)))  OR  (((TI Labelling) OR (TI Labeling) OR (TI Detection)) AND ((TI Phenomenon) OR (TI Psychological) OR (MH Absenteeism+) OR (TI Absenteeism))))  AND  ((PT "randomized controlled trial") OR (PT "controlled clinical trial") OR (TI randomized OR AB randomized) OR (TI randomised OR AB randomised) OR (TI placebo OR AB placebo) OR (TI randomly OR AB randomly) OR (TI trial OR AB trial) OR (TI groups OR AB groups) OR (MH "Epidemiologic Studies+") OR (MH "case-control studies+") OR (MH "Cohort Studies+") OR (TI "case control" OR AB "case control") OR (TI Cohort OR AB Cohort) OR (TI "Follow up" OR AB "Follow up") OR (TI Observational OR AB Observational) OR (TI Longitudinal OR AB Longitudinal) OR (TI Prospective OR AB Prospective) OR (TI Retrospective OR AB Retrospective) OR (TI Investigated OR AB Investigated) OR (TI Analysis OR AB Analysis) OR (TI Statistics OR AB Statistics) OR (TI Data OR AB Data) OR "Statistics & Numerical Data" OR "Epidemiology" OR (MH "Surveys+") OR (TI Survey OR AB Survey) OR (TI Surveys OR AB Surveys) OR (TI Questionnaire OR AB Questionnaire) OR (TI Questionnaires OR AB Questionnaires) OR (TI Study))  NOT  ((MH Animals+) NOT ((MH Animals+) AND (MH Humans+)))  NOT  ((TI "Systematic review" OR AB "Systematic review") OR (PT "Systematic Review") OR (SO "Cochrane Database Syst Rev" OR ST "Cochrane Database Syst Rev" OR IB "Cochrane Database Syst Rev") OR (PT "meta analysis") OR (TI "Meta analysis" OR AB "Meta analysis") OR (TI Meta-analysis OR AB Meta-analysis)) NOT ((MH Injections+) OR (MH Neoplasms+) OR (TI Open-Label OR AB Open-Label) OR (MH "Product Labeling+") OR (MH "Drug Labeling+") OR (MH "Affinity Labels+") OR (MH "Food Labeling+") OR (MH "Isotope Labeling+") OR (MH "Staining and Labeling+") OR (MH "In Situ Nick-End Labeling+") OR (MH "Primed In Situ Labeling+") OR (TI Placebo) OR "Drug Effects" OR (TI Drug) OR (TI Drugs) OR (TI "Food and Drug Administration") OR (TI "Food labeling") OR (TI "Calorie labeling") OR (TI Injection) OR (TI Cigarette) OR (TI Cancer) OR (TI Cancers)) |
| Embase | ((("Diagnosis" OR Diagnostic:ti,ab OR Labelling:ti,ab OR Labeling:ti,ab OR Classified:ti,ab OR Positively:ti,ab OR Diagnosis:ti,ab OR Detected:ti,ab OR Detection:ti,ab OR Scan:ti,ab)  AND  ('Mass Screening'/exp OR Screening:ti,ab OR Screened:ti,ab)  AND  ('Psychological distress':ti,ab OR 'Psychological impact':ti,ab OR 'Psychological effects':ti,ab OR 'Anxiety levels':ti,ab OR 'Anxiety Inventory' OR STAI:ti,ab OR 'Mental distress':ti,ab OR Absenteeism:ti,ab)  AND  (Patient:ti,ab OR Patients:ti,ab OR Individuals:ti,ab OR Self:ti,ab OR Parent:ti,ab OR Family:ti,ab OR Adult:ti,ab OR Men:ti,ab OR Women:ti,ab OR Children:ti,ab OR Subjects:ti,ab)  AND  (Attitude/exp OR Stigma:ti,ab OR Beliefs:ti,ab OR Well-being:ti,ab OR Wellbeing:ti,ab OR Influence:ti,ab OR Emotion:ti,ab OR Distress:ti,ab OR Mood:ti,ab OR Consequences:ti,ab OR Effect:ti,ab OR Effects:ti,ab OR Coping:ti,ab)  AND  (Before:ti,ab OR After:ti,ab OR Following:ti,ab))  OR  ((Labelling:ti OR Labeling:ti OR Detection:ti) AND (Phenomenon:ti OR Psychological:ti OR Absenteeism/exp OR Absenteeism:ti)))  AND  (term:it OR term:it OR randomized:ti,ab OR randomised:ti,ab OR placebo:ti,ab OR randomly:ti,ab OR trial:ti,ab OR groups:ti,ab OR 'Epidemiologic Studies'/exp OR 'case-control studies'/exp OR 'Cohort Studies'/exp OR 'case control':ti,ab OR Cohort:ti,ab OR 'Follow up':ti,ab OR Observational:ti,ab OR Longitudinal:ti,ab OR Prospective:ti,ab OR Retrospective:ti,ab OR Investigated:ti,ab OR Analysis:ti,ab OR Statistics:ti,ab OR Data:ti,ab OR "Statistics & Numerical Data" OR "Epidemiology" OR 'questionnaire'/exp OR Survey:ti,ab OR Surveys:ti,ab OR Questionnaire:ti,ab OR Questionnaires:ti,ab OR Study:ti)  NOT  (Animals/exp NOT (Animals/exp AND Humans/exp))  NOT  ('Systematic review':ti,ab OR term:it OR 'Cochrane Database Syst Rev':jt OR term:it OR 'Meta analysis':ti,ab OR Meta-analysis:ti,ab) NOT (Injections/exp OR Neoplasms/exp OR Open-Label:ti,ab OR 'Product Labeling'/exp OR 'Drug Labeling'/exp OR 'Affinity Labels'/exp OR 'Food Labeling'/exp OR 'Isotope Labeling'/exp OR 'Staining and Labeling'/exp OR 'In Situ Nick-End Labeling'/exp OR 'Primed In Situ Labeling'/exp OR Placebo:ti OR "Drug Effects" OR Drug:ti OR Drugs:ti OR 'Food and Drug Administration':ti OR 'Food labeling':ti OR 'Calorie labeling':ti OR Injection:ti OR Cigarette:ti OR Cancer:ti OR Cancers:ti) |
| PsycINFO | ((("Diagnosis" OR Diagnostic.ti,ab. OR Labelling.ti,ab. OR Labeling.ti,ab. OR Classified.ti,ab. OR Positively.ti,ab. OR Diagnosis.ti,ab. OR Detected.ti,ab. OR Detection.ti,ab. OR Scan.ti,ab.)  AND  (exp “Screening”/ OR Screening.ti,ab. OR Screened.ti,ab.)  AND  ("Psychological distress".ti,ab. OR "Psychological impact".ti,ab. OR "Psychological effects".ti,ab. OR "Anxiety levels".ti,ab. OR "Anxiety Inventory" OR STAI.ti,ab. OR "Mental distress".ti,ab. OR Absenteeism.ti,ab.)  AND  (Patient.ti,ab. OR Patients.ti,ab. OR Individuals.ti,ab. OR Self.ti,ab. OR Parent.ti,ab. OR Family.ti,ab. OR Adult.ti,ab. OR Men.ti,ab. OR Women.ti,ab. OR Children.ti,ab. OR Subjects.ti,ab.)  AND  (exp Attitudes/ OR Stigma.ti,ab. OR Beliefs.ti,ab. OR Well-being.ti,ab. OR Wellbeing.ti,ab. OR Influence.ti,ab. OR Emotion.ti,ab. OR Distress.ti,ab. OR Mood.ti,ab. OR Consequences.ti,ab. OR Effect.ti,ab. OR Effects.ti,ab. OR Coping.ti,ab.)  AND  (Before.ti,ab. OR After.ti,ab. OR Following.ti,ab.))  OR  ((Labelling.ti. OR Labeling.ti. OR Detection.ti.) AND (Phenomenon.ti. OR Psychological.ti. OR exp Absenteeism/ OR Absenteeism.ti.)))  AND  ("randomized controlled trial".pt. OR "controlled clinical trial".pt. OR randomized.ti,ab. OR randomised.ti,ab. OR placebo.ti,ab. OR randomly.ti,ab. OR trial.ti,ab. OR groups.ti,ab. OR exp Epidemiology/ OR "case control".ti,ab. OR Cohort.ti,ab. OR "Follow up".ti,ab. OR Observational.ti,ab. OR Longitudinal.ti,ab. OR Prospective.ti,ab. OR Retrospective.ti,ab. OR Investigated.ti,ab. OR Analysis.ti,ab. OR Statistics.ti,ab. OR Data.ti,ab. OR "Statistics & Numerical Data" OR "Epidemiology" OR Survey.ti,ab. OR Surveys.ti,ab. OR Questionnaire.ti,ab. OR Questionnaires.ti,ab. OR Study.ti.)  NOT  (exp Animals/)  NOT  ("Systematic review".ti,ab. OR "Systematic Review".pt. OR "Cochrane Database Syst Rev".jn,jw,is,it. OR "meta analysis".pt. OR "Meta analysis".ti,ab. OR Meta-analysis.ti,ab.) NOT (exp Injections/ OR exp Neoplasms/ OR Open-Label.ti,ab. OR Placebo.ti. OR "Drug Effects" OR Drug.ti. OR Drugs.ti. OR "Food and Drug Administration".ti. OR "Food labeling".ti. OR "Calorie labeling".ti. OR Injection.ti. OR Cigarette.ti. OR Cancer.ti. OR Cancers.ti.) |
| PubMed | (((Diagnosis[sh] OR Diagnostic[tiab] OR Labelling[tiab] OR Labeling[tiab] OR Classified[tiab] OR Positively[tiab] OR Diagnosis[tiab] OR Detected[tiab] OR Detection[tiab] OR Scan[tiab])  AND  ("Mass Screening"[Mesh] OR Screening[tiab] OR Screened[tiab])  AND  ("Psychological distress"[tiab] OR "Psychological impact"[tiab] OR "Psychological effects"[tiab] OR "Anxiety levels"[tiab] OR “Anxiety Inventory” OR STAI[tiab] OR "Mental distress"[tiab] OR Absenteeism[tiab])  AND  (Patient[tiab] OR Patients[tiab] OR Individuals[tiab] OR Self[tiab] OR Parent[tiab] OR Family[tiab] OR Adult[tiab] OR Men[tiab] OR Women[tiab] OR Children[tiab] OR Subjects[tiab])  AND  (Attitude[Mesh] OR Stigma[tiab] OR Beliefs[tiab] OR Well-being[tiab] OR Wellbeing[tiab] OR Influence[tiab] OR Emotion[tiab] OR Distress[tiab] OR Mood[tiab] OR Consequences[tiab] OR Effect[tiab] OR Effects[tiab] OR Coping[tiab])  AND  (Before[tiab] OR After[tiab] OR Following[tiab]))  OR  ((Labelling[ti] OR Labeling[ti] OR Detection[ti]) AND (Phenomenon[ti] OR Psychological[ti] OR "Absenteeism"[Mesh] OR "Absenteeism"[ti])))  AND  ("randomized controlled trial"[pt] OR "controlled clinical trial"[pt] OR randomized[tiab] OR randomised[tiab] OR placebo[tiab] OR randomly[tiab] OR trial[tiab] OR groups[tiab] OR "Epidemiologic Studies"[Mesh] OR "case-control studies"[Mesh] OR "Cohort Studies"[Mesh] OR "case control"[tiab] OR Cohort[tiab] OR "Follow up"[tiab] OR Observational[tiab] OR Longitudinal[tiab] OR Prospective[tiab] OR Retrospective[tiab] OR Investigated[tiab] OR Analysis[tiab] OR Statistics[tiab] OR Data[tiab] OR "statistics and numerical data"[sh] OR "epidemiology"[sh] OR "Surveys and Questionnaires"[Mesh] OR Survey[tiab] OR Surveys[tiab] OR Questionnaire[tiab] OR Questionnaires[tiab] OR Study[ti])  NOT  (Animals[Mesh] NOT (Animals[Mesh] AND Humans[Mesh]))  NOT  ("Systematic review"[tiab] OR "Systematic Review"[pt] OR "Cochrane Database Syst Rev"[ta] OR "meta analysis"[pt] OR "Meta analysis"[tiab] OR Meta-analysis[tiab])  NOT  (Injections[Mesh] OR "Neoplasms"[Mesh] OR Open-Label[tiab] OR "Product Labeling"[Mesh] OR "Drug Labeling"[Mesh] OR "Affinity Labels"[Mesh] OR "Food Labeling"[Mesh] OR "Isotope Labeling"[Mesh] OR "Staining and Labeling"[Mesh] OR "In Situ Nick-End Labeling"[Mesh] OR "Primed In Situ Labeling"[Mesh] OR Placebo[ti] OR "Drug effects"[sh] OR Drug[ti] OR Drugs[ti] OR "Food and Drug Administration"[ti] OR "Food labeling"[ti] OR "Calorie labeling"[ti] OR Injection[ti] OR Cigarette[ti] OR Cancer[ti] OR Cancers[ti]) |

Supplementary Table 6. Risk of bias of included studies: Risk of Bias in Non-Randomised Studies of Interventions (ROBINS-I).

|  | | Confounding | | Selection of participants | Classification of interventions | Deviations from intended interventions | | Missing data | Measurement of outcomes | Selection of the reported result | Overall Bias |
| --- | --- | --- | --- | --- | --- | --- | --- | --- | --- | --- | --- |
| Adriaanse 2003^1^ | |  | |  |  |  | |  |  |  |  |
| Bardi 2021^2^ | |  | |  |  |  | |  |  |  |  |
| Burton 1985^3^ | |  | |  |  |  | |  |  |  |  |
| Cheng 2006^4^ | |  | |  |  |  | |  |  |  |  |
| Cheng 2008^5^ | |  | |  |  |  | |  |  |  |  |
| Chueh 2007^6^ | |  | |  |  |  | |  |  |  |  |
| Connelly 1998^7^ | |  | |  |  |  | |  |  |  |  |
| Johnston 1984^8^ | |  | |  |  |  | |  |  |  |  |
| Jorgensen 2009^9^ | |  | |  |  |  | |  |  |  |  |
| Mann 1977^10^ | |  | |  |  |  | |  |  |  |  |
| Marteau 1991^11^ | |  | |  |  |  | |  |  |  |  |
| Quagliarini 1998^12^ | |  | |  |  |  | |  |  |  |  |
| Rimes 1999^13^ | |  | |  |  |  | |  |  |  |  |
| Rudd 1987^14^ | |  | |  |  |  | |  |  |  |  |
| Sexton 1985^15^ | |  | |  |  |  | |  |  |  |  |
| Stenn 1981^16^ | |  | |  |  |  | |  |  |  |  |
| Low | Moderate | | Serious | | | |  |  |  |  |  |

**References**

1. Adriaanse MC, Snoek FJ, Dekker JM, Spijkerman AMW, Nijpels G, Twisk JWR, et al. No substantial psychological impact of the diagnosis of type 2 diabetes following targeted population screening: the Hoorn screening study. *Diabet Med* 2004; **21**: 992-8.
2. Bardi F, Bakker M, Kenkhuis MJA, Ranchor AV, Bakker MK, Elvan A, et al. Psychological outcomes, knowledge and preferences of pregnant women on first-trimester screening for fetal structural abnormalities: a prospective cohort study. *PLoS One* 2021; **16**. Available from: https://doi.org/10.1371/journal.pone.0245938.
3. Burton BK, Dillard RG, Clark EN. The psychological impact of false positive elevations of maternal serum alpha-fetoprotein. *Am J Obstet Gynecol* 1985; **151**: 77-82.
4. Cheng PJ, Shaw SW, Lin PY, Huang SY, Soong YK. Maternal anxiety about prenatal screening for group B streptococcus disease and impact of positive colonization results. *Eur J Obstet Gynecol Reprod Biol* 2006; **128**: 29-33.
5. Cheng PJ, Wu TL, Shaw SW, Chueh HO, Lin CT, Hsu JJ, et al. Anxiety levels in women undergoing prenatal maternal serum screening for Down syndrome: the effect of a fast reporting system by mobile phone short-message service. *Prenat Diagn* 2008; **28**: 417-21.
6. Chueh HY, Cheng PJ, Shaw SW, Lin CT, Hsu JJ, Hsieh TT. Maternal anxiety about first trimester nuchal translucency screening and impact of positive screening results. *Acta Obstet Gynecol Scand* 2007; **86**: 1437-41.
7. Connelly J, Cooper J, Mann A, Meade TW. The psychological impact of screening for risk of coronary heart disease in primary care settings. *J Cardiovasc Risk* 1998; **5**: 185-91.
8. Johnston ME, Gibson ES, Terry CW. Effects of labelling on income, work and social function among hypertensive employees. *J Chronic Dis* 1984; **37**: 417-23.
9. Jørgensen T, Ladelund S, Borch-Johnsen K, Pisinger C, Schrader AM, Thomsen T, et al. Screening for risk of cardiovascular disease is not associated with mental distress: the Inter99 study. *Prev Med* 2009; **48**: 242-6.
10. Mann AH. The psychological effect of a screening programme and clinical trial for hypertension upon the participants. *Psychol Med* 1977; **7**: 431-8.
11. Marteau TM, Cook R, Kidd J, Michie S, Johnston M, Slack J, et al. The psychological effects of false-positive results in prenatal screening for fetal abnormality: a prospective study. *Prenat Diagn* 1992; **12**: 205-14.
12. Quagliarini D, Betti S, Brambati B, Nicolini U. Coping with serum screening for Down syndrome when the result is given as a numeric value. *Prenat Diagn* 1998; **18**: 816-21.
13. Rimes KA, Salkovskis PM, Shipman J. Psychological and behavioural effects of bone density screening for osteoporosis. *Psychol Health* 1999; **14**: 585-608.
14. Rudd P, Price MG, Graham LE, Beilstein BA, Tarbell SJ, Bacchetti P, et al. Consequences of worksite hypertension screening: changes in absenteeism. *Hypertens* 1987; **10**: 425-36.
15. Sexton M, Schumann BC. Sex, race, age, and hypertension as determinants of employee absenteeism. *Am J Epidemiol* 1985; **122**: 302-10.
16. Stenn PG, Noce A, Buck C. A study of the labelling phenomenon in school children with elevated blood pressure. *Clin Invest Med* 1981; **4**: 179-81.

Supplementary Table 7. Clinical meaningfulness of outcome measures.

|  | Questionnaire | Score range | High score meaning | Clinical Range |
| --- | --- | --- | --- | --- |
| Anxiety | STAI^1^ | 20-80 | Higher anxiety | >40 |
|  | SCL-90-R Anxiety subscale^2^ | 0-4 | Higher anxiety | 0.75 – moderately symptomatic  1.35 – severely symptomatic |
|  | VAS-A^3^ | 0-100 | Higher anxiety | >53.2^a^ |
| Depression | SCL-90-R Depression subscale^2^ | 0-4 | Higher depression | 0.73 – moderately symptomatic  1.50 – severely symptomatic |
|  | VAS-D^3^ | 0-100 | Higher depression | >51.3^a^ |
| Wellbeing | GHQ^4 5 6^  28-item version  30-item version | 0-28  0-30 | Lower general wellbeing | <4/5^b^  <3^b^ |
|  | W-BQ12^7^ | 0-36 | Higher general wellbeing | Unavailable |

*Note.* STAI = State Trait Anxiety Inventory; SCL-90-R = Symptom Checklist 90 revised; VAS-A = Visual Analogue Scale for Anxiety; VAS-D = Visual Analogue Scale for Depression; GHQ = General Health Questionnaire; W-BQ12 = Wellbeing Questionnaire 12 item; ^a^Cut-off has been scaled up from a 0-10 VAS to a 0-100 VAS scale to align with measurement in included study; ^b^GHQ, not Likert, scoring used.

**References**

1. Spielberger CD, Gorsuch RL, Lushene PR, Vagg PR, Jacobs GA. *Manual for the state-trait anxiety inventory*. Consulting Psychologists Press Inc, 1983.
2. Derogatis LR. *SCL-90-R: symptom checklist-90-R. Administration, scoring and procedures manual* 3rd edn. National Computer Systems, 1994.
3. Lesage FX, Berjot S, Deschamps F. Clinical stress assessment using a visual analogue scale. Occup Med 2012; **62**; 600-5.
4. Goldberg D, Williams P. *A user’s guide to the general health questionnaire*. NFER Nelson, 1988.
5. Goodchild M, Duncan-Jones P. Chronicity and the general health questionnaire. *B J Psych* 1985; **146**: 55-61.
6. Mann AH. The psychological effect of a screening programme and clinical trial for hypertension upon the participants. *Psychol Med* 1977; **7**: 431-8.
7. Bradley C. The well-being questionnaire. In *Handbook of psychology and diabetes: a guide to psychological measurement in diabetes research and practice* (ed C Bradley): 89-109. Harwood Academic 1994.

Supplementary Figure 1. Mean change in state anxiety scores from baseline to immediate follow-up: post-hoc sensitivity analysis.


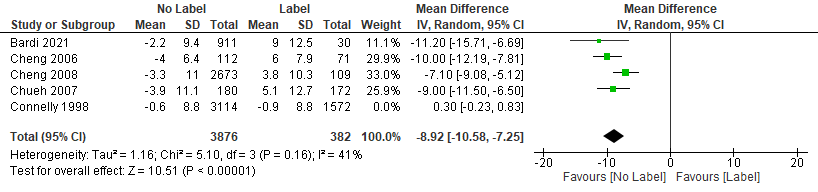


Supplementary Figure 2. Mean change in state anxiety scores from baseline to immediate follow-up: additional post-hoc sensitivity analysis.


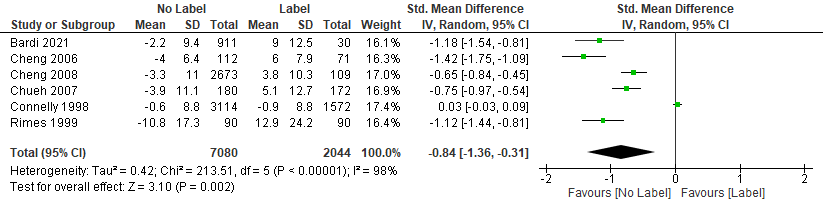


Supplementary Figure 3. Mean change in state anxiety scores from baseline to immediate follow-up: additional post-hoc sensitivity analysis (one study from planned analysis removed).


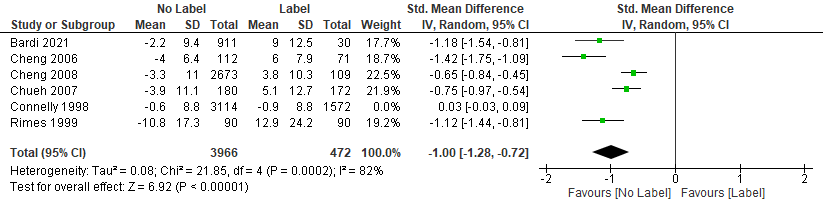


Supplementary Figure 4. Mean change in state anxiety scores from baseline to longer-term follow-up.


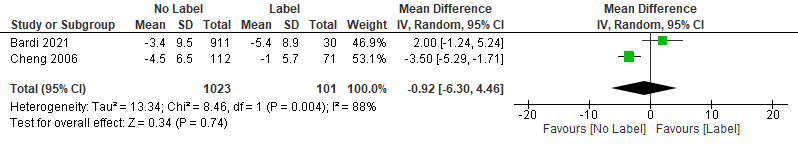


Supplementary Figure 5. Mean change in state anxiety scores from baseline to longer-term follow-up: fixed effects analysis.


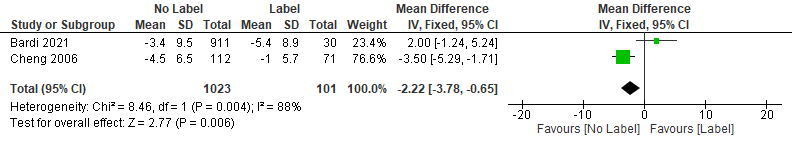


Supplementary Figure 6. Mean change in state anxiety scores from baseline to longer-term follow-up: post-hoc sensitivity analysis with additional study.


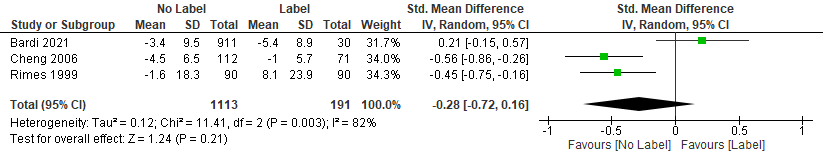


Supplementary Figure 7. Mean change in state anxiety scores from baseline to longer-term follow-up: post-hoc sensitivity analysis with additional study (fixed effects analysis).


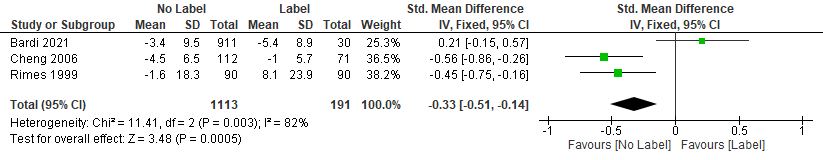


Supplementary Figure 8. Mean change in depression scores from baseline to immediate follow-up.

Supplementary Figure 9. Mean change in general mental health scores from baseline to immediate follow-up.


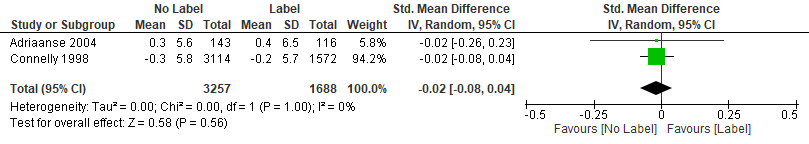


Supplementary Figure 10. Mean change in absenteeism from year prior to year following screening.


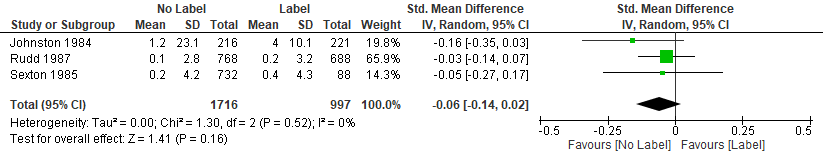


Supplementary Figure 11. Mean change in anxiety scores at baseline, immediate follow-up, and three-month follow-up.
